# Expression of the anti-apoptotic BAG3 protein in leg venous ulcerative tissues

**DOI:** 10.1038/cddiscovery.2015.68

**Published:** 2016-01-25

**Authors:** N Campitiello, M Faenza, D Pagliara, C Baldi, P Zeppa, A Rosati, C Rubino

**Affiliations:** 1 ‘SS. Giovanni di Dio e Ruggi d’Aragona—Schola Medica Salernitana’, University of Salerno, Salerno, Italy; 2 Department of Medicine and Surgery, School in Translational Medicine, University of Salerno, Baronissi, Salerno, Italy; 3 BIOUNIVERSA s.r.l., Baronissi, Salerno, Italy

**To the Editor**

The healing process after an injury consists of a complex cascade of cellular and molecular events; the failure of one or more of these events causes chronic tissue ulceration that, despite several therapeutic options, represents a significant problem in our society.^1^ Notably, clinical examination of wounds is not sufficient in predicting which wounds will have impaired ability to heal and which treatment will succeed. In this respect, the identification of molecules involved in this process is needed for improving our management of repair.^2,3^ BAG3, a stress response- induced co-chaperone, interacts with Hsp70 and other proteins through its BAG, WW, proline-rich and IPV(Ile-Pro-Val) motifs; it regulates several cellular processes including apoptosis, autophagy and cell motility.^4–6^ Although its participation in stress response may suggest a possible role for BAG3 in the healing pathologies, BAG3 expression in ulcerated wounds had never been studied.

To this end, BAG3 expression was analyzed by immunohystochemistry in ulcerous tissues from 13 patients with lower leg venous ulcers of different etiology (diabetes, venous insufficiency, trauma or vasculitis). BAG3 staining resulted positive in a portion of the tissue analyzed (Figure 1a) and its highest levels were found in endothelial and stroma cells in the granulation tissues (Figures 1a and b). Notably, endothelial cells in normal skin adjacent to ulcerated tissue resulted negative (Figure 1b, right panel). Because BAG3 was not expressed in all the analyzed tissues, a possible association between BAG3 expression and specific features of tissues, possibly reflecting specific phases of the healing process, were analyzed (Figure 1c). We found a complete association (*P*=0.001) between BAG3 and presence of granulation tissue. Indeed, all the nine specimens with granulation tissue features were positive for BAG3 expression; on the other hand, specimens in which granulation tissue was not detectable were BAG3 negative. Therefore, BAG3 appeared expressed in the proliferation/repair phase of wound healing, probably sustaining cell survival and/or cell cycle progression. Consistently, the presence of endothelial hyperplasia in the granulation tissue was significantly (*P*=0.021) associated with BAG3 expression. BAG3 positivity of neo-vessels in granulation tissues is in agreement with the reported expression of BAG3 in tumor neoangiogenetic vessels.^7^ Importantly, this finding confirms the link between BAG3 expression and neoangiogenesis in a different pathophysiological context, probably in connection with BAG3 role in regulating ERK-DUSP6 interaction and cell cycle progression.^7^ In addition, BAG3 expression was observed not only in neo-endothelial but also in stromal cells. Therefore, BAG3 might also participate in other stromal cell activities that contribute to skin repair. The identification of BAG3-positive stromal cellular components, and investigation of BAG3 role in their functional activities (survival/proliferation, migration and cytokine production) could contribute to our understanding of physiological and pathological mechanisms in tissue repair.^1–3^

## Figures and Tables

**Figure 1:**
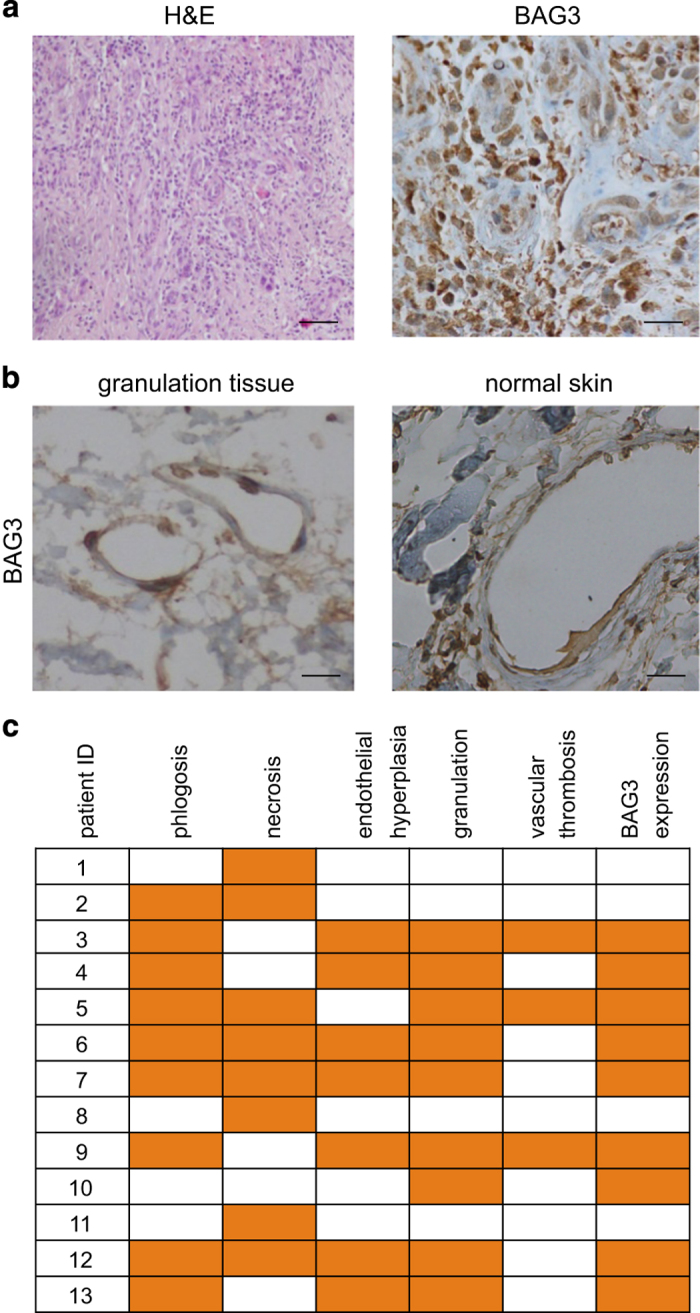
BAG3 expression in ulcerous tissues. Tissues were obtained from 13 patients with lower leg venous ulcers (median age±s.d. is 70.6±10.6; six male and seven female subjects). The ethics committee of Azienda Ospedaliera Ospedali Riuniti S. Giovanni e Ruggi d’Aragona di Salerno approved this study and informed consent was obtained from all patients. Immunohistochemistry was performed using the anti-BAG3 monoclonal antibody AC-1 (BIOUNIVERSA s.r.l., Salerno, Italy). In brief, IHC protocol included deparaffination in xylene, rehydration through descending degrees of alcohol up to water, incubation with 3% hydrogen peroxidase for 5 min to inactivate endogenous peroxidases and non-enzymatic antigen retrieval in citrate buffer, pH 6.0, for 30 min at 95 °C. Samples were then rinsed, blocked with 5% fetal bovine serum in 0.1% PBS/BSA and then incubated for 1 h at room temperature with the anti-BAG3 mAb in saturating conditions. The standard streptavidin-biotin-linked horseradish peroxidase technique was then performed, and 3,3′-diaminobenzidine was used as a substrate chromogen solution. Finally, the sections were counterstained with hematoxylin; slides were then coverslipped using a synthetic mounting medium. (**a**) Granulation tissue in an ulcerous sample. H&E, Hematoxylin and eosin staininig (bar, 100 *μ*m); *BAG3*, BAG3 staining by IHC (bar, 30 *μ*m). Similar images were obtained in the other eight ulcerous samples showing granulation tissue features. (**b**) BAG3 staining in endothelial cells in ulcerous tissue and normal skin (bar, 30 *μ*m). (**c**) Orange boxes were used to indicate the presence of a histologial feature and BAG3 expression in the ulcerous tissues analyzed. Statistical associations were calculated by using Fisher’s exact test in 2×2 contingency tables.
